# Detection of Rare Somatic *GNAS* Mutation in McCune-Albright Syndrome Using a Novel Peptide Nucleic Acid Probe in a Single Tube

**DOI:** 10.3390/molecules22111874

**Published:** 2017-11-01

**Authors:** Fu-Sung Lo, Tai-Long Chen, Chiuan-Chian Chiou

**Affiliations:** 1 Division of Pediatric Endocrinology & Genetics, Chang Gung Memorial Hospital, Linkou, Taoyuan 333, Taiwan; lofusu@cgmh.org.tw; 2 School of Medicine, College of Medicine, Chang Gung University, Taoyuan 333, Taiwan; 3 Molecular Medicine Research Center, College of Medicine, Chang Gung University, Taoyuan 333, Taiwan; tl.chen@outlook.com; 4 Department of Medical Biotechnology and Laboratory Science, College of Medicine, Chang Gung University, Taoyuan 333, Taiwan; 5 Department of Thoracic Medicine, Chang Gung Memorial Hospital, Linkou, Taoyuan 333, Taiwan

**Keywords:** peptide nucleic acid probe, sensitive detection, McCune-Albright syndrome, *GNAS* mutation

## Abstract

McCune-Albright syndrome (MAS) is characterized by the triad of precocious puberty, café au lait pigmentation, and polyostotic fibrous dysplasia (FD) of bone, and is caused by post-zygotic somatic mutations—R201H or R201C—in the guanine nucleotide binding protein, alpha stimulating (*GNAS*) gene. In the present study, a novel peptide nucleic acid (PNA) probe with fluorescent labeling was designed to detect trace amounts of somatic mutant *GNAS* in a single tube reaction. The method was applied to screen *GNAS* mutations in six patients with MAS/FD. The results showed that the PNA probe assay could detect low abundant mutants in 200-fold excess of wild-type alleles. The *GNAS* mutation was found in three patients with severe disease (MAS) by using the assay. The other three patients with mild disease (having only FD) showed a wild-type result. This study has provided a simple method to detect trace amounts of *GNAS* mutants with high sensitivity in large amounts of wild-type DNA.

## 1. Introduction

McCune-Albright syndrome (MAS) and fibrous dysplasia (FD) are believed to be caused by postzygotic somatic mutations in the alpha subunit of the stimulatory G-protein (Gsα), which is encoded by *GNAS1* gene. The most frequently reported mutations are R201H or R201C [1,2,3], which result in a loss of the GTPase activity of Gsα and lead to constitutive activation of Gsα. Cyclic adenosine monophosphate is thus accumulated in the affected cells and disturbs normal cell function [4]. The mechanism of these somatic mutations of *GNAS1* gene is not yet known. Because the *GNAS1* mutations occur at different life stages, the genotypes of *GNAS1* in different patients are mosaic, with different ratio of wild-type and mutant. The ratio and distribution of the mutant cells lead to a broad spectrum of diseases. The mild forms include monostotic FD and polyostotic FD; the most severe form is MAS, which is characterized by at least two of the following features: precocious puberty, café au lait pigmentation, and polyostotic FD [5]. MAS is a rare disease, with an estimated prevalence between 1/100,000 and 1/1,000,000.

MAS is usually diagnosed by applying bone biopsy and imaging methods such as computed tomography or magnetic resonance imaging. However, as the disease symptoms are diverse, molecular testing of *GNAS* mutation is helpful to establish the diagnosis. DNA samples for detecting *GNAS* mutations can be isolated from the skin [6], whole blood [5,7], and fibrous dysplasia lesions [2,8] of patients. Among them, blood is an especially useful source of the sample. Obtaining DNA from blood cells avoids the requirement of many invasive procedures, such as bone biopsy. However, the mosaic pattern of cells bearing the *GNAS* mutation may hamper the detection, as mutant *GNAS* alleles usually represent only a small proportion of the total number of *GNAS* alleles. The possibility of detection is proportional to the severity of the disease. Hence, a sensitive molecular test will increase the detection rate.

Peptide nucleic acid (PNA) is a DNA mimic in which the deoxyribose phosphate backbone is replaced with a 2-aminoethylglycine backbone. A PNA oligomer can form a stable Watson–Crick pairing with complementary DNA, but the pairing is highly sensitive to mismatches [9]. Two different designs of PNA oligomer have been reported to detect *GNAS* mutations. The first is to use PNA as a clamp in the polymerase chain reaction (PCR) to inhibit the wild-type amplification [10,11,12]. Rare mutant PCR products are thus enriched, and can be analyzed by gel electrophoresis and direct sequencing. The second design is to label PNA with a fluorophore, serving as a sensor probe. It has been used to quantify mutant *GNAS* by melting profile [13]. However, the PNA sensor probe was added after PCR reaction, which needed multiple manipulation steps and increased contamination risk. In addition, this method was much less sensitive.

**Figure 1:**
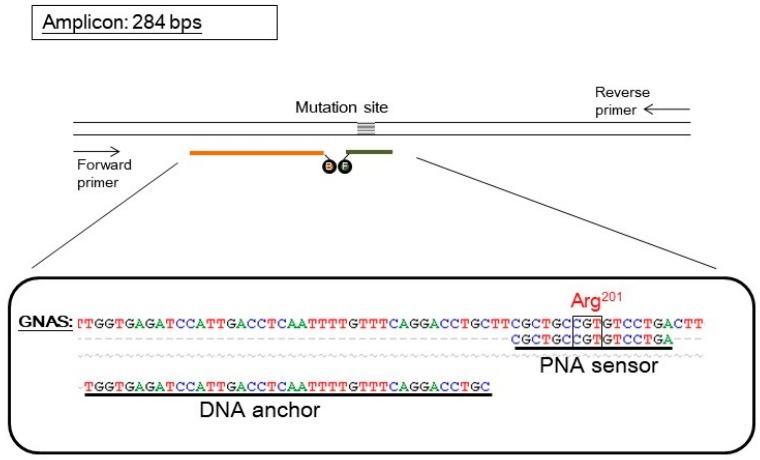
The design of a PNA probe assay for detecting *GNAS* mutation. The upper part shows the scheme of oligonucleotides. The parallel lines represent a double-stranded DNA fragment of the *GNAS* gene. The pair of arrows represents the forward and reverse primers used in PCR that amplified the *GNAS* gene and generated an amplicon of 284 base pairs (bps). The probe set consisted of a PNA sensor probe (orange line) which covered the mutation site and was labeled with a fluorescein (represented by an “F”) and a DNA anchor probe (dark green line) labeled with Bodipy 630/650 (represented by a “B”). The two fluorophores can undergo Foster resonance energy transfer when both probes anneal on the same target *GNAS* fragment. The lower part shows the sequence alignment of wild-type *GNAS*, the PNA sensor probe, and the DNA anchor probe. The mutation site (arg201) is also indicated. For simplicity, the fluorophores are not shown.

In the current study, we combined both designs to develop a simple reaction for detecting *GNAS* mutation (see Figure 1 for a schematic diagram). The fluorescence-labeled PNA was used not only to clamp wild-type amplification, but also to report mutant signal. This design has been successfully applied in the detection of *KRAS* mutations [14,15]. With this design, the enrichment of mutant PCR products and the detection of mutant genotype can be completed in a single tube without further laborious procedures. We used this novel PNA probe assay to screen *GNAS* mutation in blood samples of six patients with polyostotic FD/MAS and identified three with a *GNAS* mutation.

## 2. Results

### 2.1. Assay Design

The PNA probe assay for *GNAS* mutation contained a pair of hybridization probes and a pair of PCR primers. A PNA oligomer was labeled with a fluorescein, and served as both PCR clamp and sensor probe. A DNA oligomer was labeled with a Bodipy 630/650, and served as an anchor probe. The pair of primers generated an amplicon of 284 base pairs (Figure 1). The PNA sequence was designed according to the wild-type *GNAS*; hence, it is perfectly complementary to the wild-type allele but has a mismatch to the mutant allele of *GNAS*.

**Figure 2:**
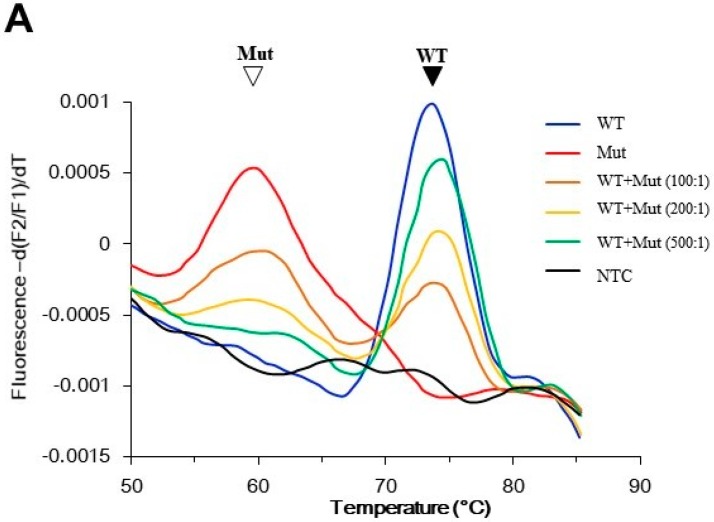

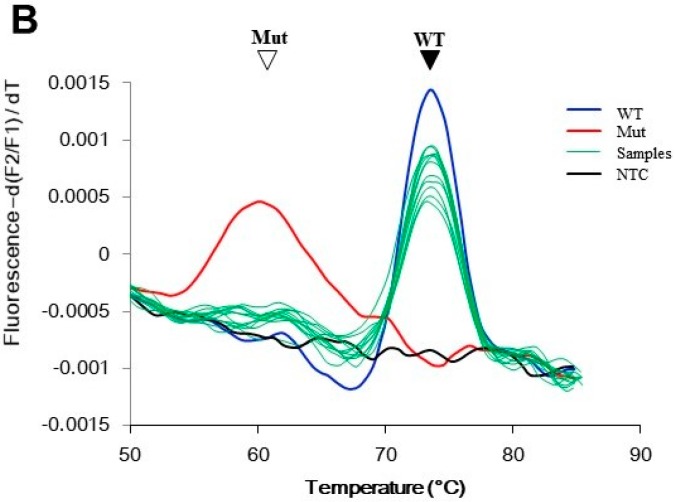
Performance of the PNA probe assay. The assay generated melting peaks of the PNA/*GNAS* duplex. The PNA probe was perfectly matched with the wild-type *GNAS*, and hence had a peak at higher melting temperature (*T*_m_) than the peak from the mismatched mutant *GNAS.* (**A**) Assay sensitivity was determined using control templates. The indicated ratio of mutant and wild-type plasmid DNA were mixed, and the mutant was detected by PNA probe assay. The filled arrowhead indicates the wild-type peak; the open arrowhead indicates the mutant peak; (**B**) Specificity of the assay. DNA (100 ng) from peripheral blood of 20 individuals without McCune–Albright syndrome/fibrous dysplasia (MAS/FD) were analyzed by the PNA probe assay. Only wild-type melting peaks were observed in these samples (green lines). The control reactions were conducted using either 10 pg wild-type plasmid DNA (blue line), 0.1 pg mutant plasmid DNA (red line), or no template (black line). Mut: mutant; NTC: no template control; WT: wild-type.

The assay procedure included a PCR program and a melting analysis program on a capillary PCR instrument with a fluorescent detector. During the PCR program, the PNA bound tightly to the wild-type template and hindered its amplification. On the other hand, because of the existence of a mismatch, the PNA bound to the mutant template much more loosely and allowed its amplification. As a consequence, the PNA served as a PCR clamp for wild-type templates, which enriched the mutant products in PCR. During the melting analysis program, the fluorescein on the PNA sensor probe could undergo Forster resonance energy transfer (FRET) with the Bodipy on the DNA anchor probe when both probes were annealed to the *GNAS* fragment. Measuring the FRET fluorescence along with temperature change revealed the association–dissociation status between the PNA sensor probe and the *GNAS* sequence. Further, plotting the negative derivative of the fluorescence over temperature (-dF/dT) against temperature generated melting peaks (as shown in Figure 2). The PCR products derived from mutant templates had a lower melting temperature (mutant *T*_m_) than that from wild-type templates (wild-type *T*_m_), so mutation can be identified through the existence of the mutant melting peak. Therefore, the assay can be completed in a single tube without the need for further manipulation.

In a preliminary experiment using control templates, the wild-type DNA generated a peak at 75 °C, and the mutant DNA at 60 °C. Rare mutant alleles could reveal a mutant peak even in a 200-fold excess of wild-type alleles. In addition, the size of the mutant peak was positively correlated with the amount of mutant allele present. Hence, the assay was also semi-quantitative (Figure 2A). To demonstrate the assay specificity, blood samples from 20 healthy individuals were analyzed, and they all showed wild-type results (Figure 2B).

### 2.2. Genotypes Detected by the PNA Probe Assay

**Figure 3:**
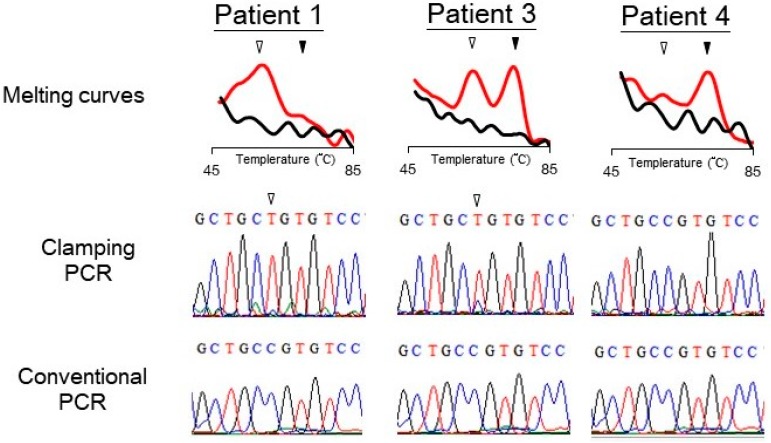
Typical results of detecting *GNAS* mutation in three patients with suspect McCune–Albright syndrome or fibrous dysplasia. The PNA probe assay generated melting curves of PCR products of the samples (top). The PCR products (marked as clamping PCR) were analyzed by Sanger sequencing (middle). For comparison, sequencing results from PCR products without using PNA probe (marked as conventional PCR) are also shown (bottom). Open arrowheads indicate the position of mutant bases or mutant peaks; filled arrowheads indicate the wild-type peaks.

DNA samples from six patients with MAS/FD were analyzed for *GNAS* mutation by the PNA probe assay. The existence of mutation was determined through the melting peak profile. The PNA probe assay identified three patients with mutant *GNAS* (Table 1). To further demonstrate that the mutant melting peaks came from a disease-causing genotype, the PCR products were sent to Sanger sequencing analysis (shown as “clamping PCR” in Figure 3), which confirmed that these “expected mutants” had a point mutation at codon 201. For comparison, the samples were also amplified with conventional PCR without the PNA probe (conventional PCR). Sequencing of the conventional PCR products detected no mutation in the samples. Figure 3 shows the melting and sequencing results from three of the patients. In this figure, Patients 1 and 3, but not Patient 4, have an R201 mutation. Of the six samples, three (Patients 1–3) from patients with more severe symptoms (having both precocious puberty and FD) had a mutant *GNAS*. Clinical features of Patient 1 are shown in Supplemental Figure S1. The other three (Patient 4–6) from patients with mild symptoms (only having FD) showed wild-type results (Table 1 and Supplemental Figure S2).

## 3. Discussion

We have demonstrated the application of a single tube reaction using PNA as both PCR clamp and sensor probe for the detection of *GNAS* mutation. Unlike other reported methods that used PNA only for PCR clamping and needed to check the sequences of PCR products, our newly developed method couples PCR amplification, mutant enrichment, and mutation detection; hence, the assay can be accomplished in a single tube on a real-time PCR instrument without the need to go through several laborious procedures such as electrophoresis, hybridization, and enzymatic reaction.

The establishment of the PNA probe assay that can detect rare mutants in large amounts of wild-type DNA is difficult. The key point is clamping efficiency of the PNA probe in wild-type amplification, which reduces the amount of the wild-type products and enriches the mutant products during PCR. In the end of PCR, the wild-type products must be less than the mutant products; otherwise, it would mask the mutant peak in the melting analysis. To optimize the clamping efficiency, several factors are important: (1) The *T*_m_ of the PNA/wild-type duplex needs to be located between 65 °C and 75 °C; (2) The extension temperature in PCR needs to be around 10 °C lower than the wild-type *T*_m_ mentioned above, so that the PNA can clamp the wild-type amplification efficiently. For example, the extension temperature of our *GNAS* assay was 60 °C, which was 13 °C lower than the wild-type *T*_m_ at 73 °C; (3) The distance between the binding sites of the forward primer and the PNA probe must be greater than 90 nucleotides, which prevent the *Taq* DNA polymerase from running off the wild-type templates during temperature ramping (for detailed explanation, please refer to References [14,15]). (4) Asymmetric PCR can enhance the clamping efficiency. In our *GNAS* assay, the concentration of the forward primer was one fifth that of the reverse primer.

A major concern regarding the highly sensitive method is its specificity or false positive rate. Several facts reveal that our method is specific: Firstly, during the stage of method development, we did not find any mutant melting peaks in negative control using wild-type DNA (not shown); Secondly, we tested our method in twenty DNA samples from individuals without MAS/FD, which did not show any false positive result (Figure 2); Finally, the PCR products showing mutant melting peaks were sequenced to confirm the R201C mutation type (Figure 3). If the mutant melting peak came from a PCR error or other non-specific amplification, sequencing results would more likely be random variations, but not a specific mutant genotype. Note that the sequencing procedure in Figure 3 was not necessary for the assay. It was just for re-confirming the melting results.

In contrast to assay specificity, assay sensitivity is another important issue to evaluate. Although we have demonstrated that our assay had a comparable analytical sensitivity to some other PNA methods (see Reference [11] for an example), clinical sensitivity is not easy to measure on the MAS patients. One reason is that MAS is a rare disease, and it is very difficult to obtain a sufficient number of positive samples for statistical analysis. Another reason is that mutant-bearing cells in MAS/FD patients are unevenly distributed and highly mosaic, so the concentrations of mutant cells in the blood vary according to disease severity. Our results showing that only patients with severe symptoms (Patient 1–3) had detectable *GNAS* mutation confirm this observation. Since the assay is not a yes-or-no detection, the patients with mild symptoms (Patients 4–6) may also have *GNAS* mutation, but the level of released mutant cells in peripheral blood is too few to be detected. A previous studies had similar observation that only a part of the MAS/FD patients had detectable *GNAS* mutations in peripheral blood [11].

In the three MAS patients, two of them were a pair of monozygotic (MZ) twins (Patients 1 and 2, Table 1). The twins were almost concordant for MAS: both had precocious puberty and polyostotic fibrous dysplasia, except lacking skin lesion. To our knowledge, no MZ twins have been reported to be completely concordant for MAS. Usually only one of the twins had typical MAS, while the other had no symptom or had only mild bone lesions or endocrinopathy [16,17,18]. The discrepancy may be due to the timing of postzygotic mutation that influences the distribution and phenotypic effect of mutant cells in the individual [19]. If this occurs at the embryonic stem cell stage or the inner cell mass stage, tissues from all three germ layers will be affected, and the MAS phenotypes emerge [20]. However, there might be some other inherited or environmental factors involved in the disease, making one or both MZ twins affected.

MAS/FD is a rare disease, so the test of *GNAS* mutation is not frequently performed in clinical laboratories. However, recent studies have shown that a pancreatic cancer lesion—intraductal papillary mucinous neoplasm (IPMN)—bears *GNAS* mutation [21]. As the IPMN shows relatively benign characteristics and is not yet invasive cancer, diagnosis of IPMN increases the opportunity for curative therapies. In addition, *GNAS* mutations are also involved in hepatocellular carcinoma [22], kidney cancer [23], and colorectal tumors [24]. These findings will extend the applicability of our PNA probe assay. In addition, the PNA probe design can be modified in the detection of other somatic mutations, such as *EGFR*, *KRAS*, or *BRAF* mutations in many cancers.

## 4. Materials and Methods

### 4.1. Design of Probes and Primers

A forward primer and a reverse primer were designed to amplify *GNAS* fragments in exon 8. The sensor probe covering the variable region is a 16-mer PNA labeled with a fluorescein at the N-terminus (equivalent to the 5′-end of a DNA oligomer). The anchor probe is a 40-mer DNA labeled with fluorescent dye Bodipy 630/650 at the 3′-end via an OO linker. The design of the primers and the probes were according to a guideline described elsewhere [14]. PCR primers and the anchor probe were provided by TIB MOLBIOL (Berlin, Germany). PNA was provided by Applied Biosystems (Forster City, CA, USA). Sequences of primers and probes used in this study are listed in Table 2.

### 4.2. Patients

This study was approved by the Institutional Review Board of Chang Gung Memorial Hospital (with approval number 101-0650A3). After obtaining informed consent, three patients with MAS and three with only FD of bone were screened for mutations of *GNAS* gene in blood. Patients with MAS have characteristics of precocious puberty, café au lait pigmentation, and polyostotic fibrous dysplasia of bone. Patients with only FD of bone do not show precocious puberty. Clinical characteristics of the three patients with MAS are summarized in Supplemental Materials Table S1.

### 4.3. Preparation of Template DNA

Sample DNA was extracted from 200-μL aliquots of whole blood (with EDTA as anticoagulant) using a QIAamp DNA-blood-mini kit (Qiagen). One hundred ng of the eluted DNA was used as PCR template. The control templates were plasmids with the wild-type or the mutant DNA fragment as an insert. Mutant plasmid DNA (0.1 pg) was added into different amounts of wild-type plasmid DNA, generating 100–500 fold wild-type backgrounds. The assay was performed under the conditions described below.

### 4.4. Detection of GNAS Mutation

The PCR mixture (20 μL) contained 50 mM Tris (pH 8.5), 3 mM MgCl2, 500 μg/mL BSA, 200 μM of each deoxyribonucleoside triphosphate, 0.2 μM forward, 1 μM reverse primers, 0.5 μM PNA sensor probe, 0.5 μM DNA anchor probe, 0.5 U Platinum *Taq* (ThermalFisher Scientific), and templates. The assay was performed on a LightCycler 1.5 or 2.0 (Roche Diagnostics, Mannheim, Germany). The thermal program contained an amplification step and a melting step. The amplification step started with a 3-min denaturation at 95 °C, then ran for 55 cycles as follows: 95 °C for 5 s, 53 °C for 3 s, and 60 °C for 20 s. The melting step was performed after a 30 s denaturation at 95 °C and then decreasing the temperature to 40 °C at ramp rate 0.7 °C/s. The fluorescent signal was detected in channel F2 for the BODIPY labeled probes. To confirm results and determine specific mutation types, PCR products were separated on a 2% agarose gel, eluted, and then sequenced by an automated DNA sequencer.

## 5. Conclusions

Our study indicated that PNA was not only a good clamp in PCR for enriching rare mutants but also a superior sensor probe for genotyping. Both features make it possible to create a simple and sensitive method to detect trace amounts of *GNAS* mutants in a large amount of wild-type DNA. This method has great potential for the screening of diseases with *GNAS* mutation.

## Figures and Tables

**Table 1:** Clinical characteristics of six patients with MAS or FD.

Patient		Sex	Age (Year)	PP ^1^	Café-au-lait Spots	BFD ^2^	*GNAS* Mutations
1	twin A	Female	0.39	+	+	+	+
2	twin B	Female	7.83	+	−	+	+
3		Female	5.28	+	−	+	+
4		Female	11.95	−	+	+	−
5		Male	12.05	−	−	+	−
6		Male	6.25	−	−	+	−

^1^ PP: precocious puberty; ^2^ BFD: bone fibrous dysplasia.

**Table 2:** Primers and probes used in this study.

Name	Sequence (5′-3′ for DNA or N Terminal to C Terminal for PNA)	Length
Primers		
Forward	AACTACTCCAGACCTTTGCTTTAGAT	26
Reverse	CAGCTGGTTATTCCAGAGGGAC	22
Probes		
PNA sensor	(Fluorescein)-OO-CGCTGCCGTGTCCTGA	16
DNA anchor	TGGTGAGATCCATTGACCTCAATTTTGTTTCAGGACCTGC-(Bodipy630/650)	40

## Supplementary Materials

The following are available online. Table S1 Clinical and laboratory findings of three patients with McCune-Albright syndrome. Figure S1 Features of Patient 1. Figure S2 Melting curves of all patients.
