# Supplementary material for: Detection of Rare Somatic GNAS Mutation in McCune-Albright Syndrome Using a Novel Peptide Nucleic Acid Probe in a Single Tube
Source: Molecules. 2017 Nov 1;22(11):1874. doi: 10.3390/molecules22111874 (PMC6150203; doi:10.3390/molecules22111874)
Supplement: Supplementary file 1 [file molecules-22-01874-s001.pdf]

Supplemental Table S1. Clinical and laboratory findings of three patients with McCune-Albright syndrome.

	Patient 1(Twin B)	Patient 2 (Twin A)	Patient 3
Sex	Female	Female	Female
Age (year)	0.39	7.83	5.28
Bone Age (year)	0.25	8.83	5.75
Height (cm)	52	129.2	114.6
Height SDS[a]	-3.85	0.81	0.91
Weight (kg)	6.8	35.3	23
Weight SDS	-0.08	1.7	1.35
BMI (kg/m^2)	25.15	21.15	17.51
BMI SDS[a]	5.1	1.81	1.08
Target height (cm)	159	159	164.5
Target height SDS[a]	-0.04	-0.04	0.95
Gestation Age (week)	38	38	40
Birth weight (gm)	1900	1800	2900
Café au lait pigmentation	+	-	-
Precocious puberty	+	+	+
Bone dysplasia	+	+	+
FSH (mIU/mL) baseline	<0.5	0.4	1.24
FSH (mIU/mL) peak		0.9	1.87
LH (mIU/mL) baseline	<0.5	<0.1	0.53
LH (mIU/mL) peak		<0.1	0.65
E2 (pg/mL) baseline	93.1	43.26	44.01

[a]SDS, standard deviation score.

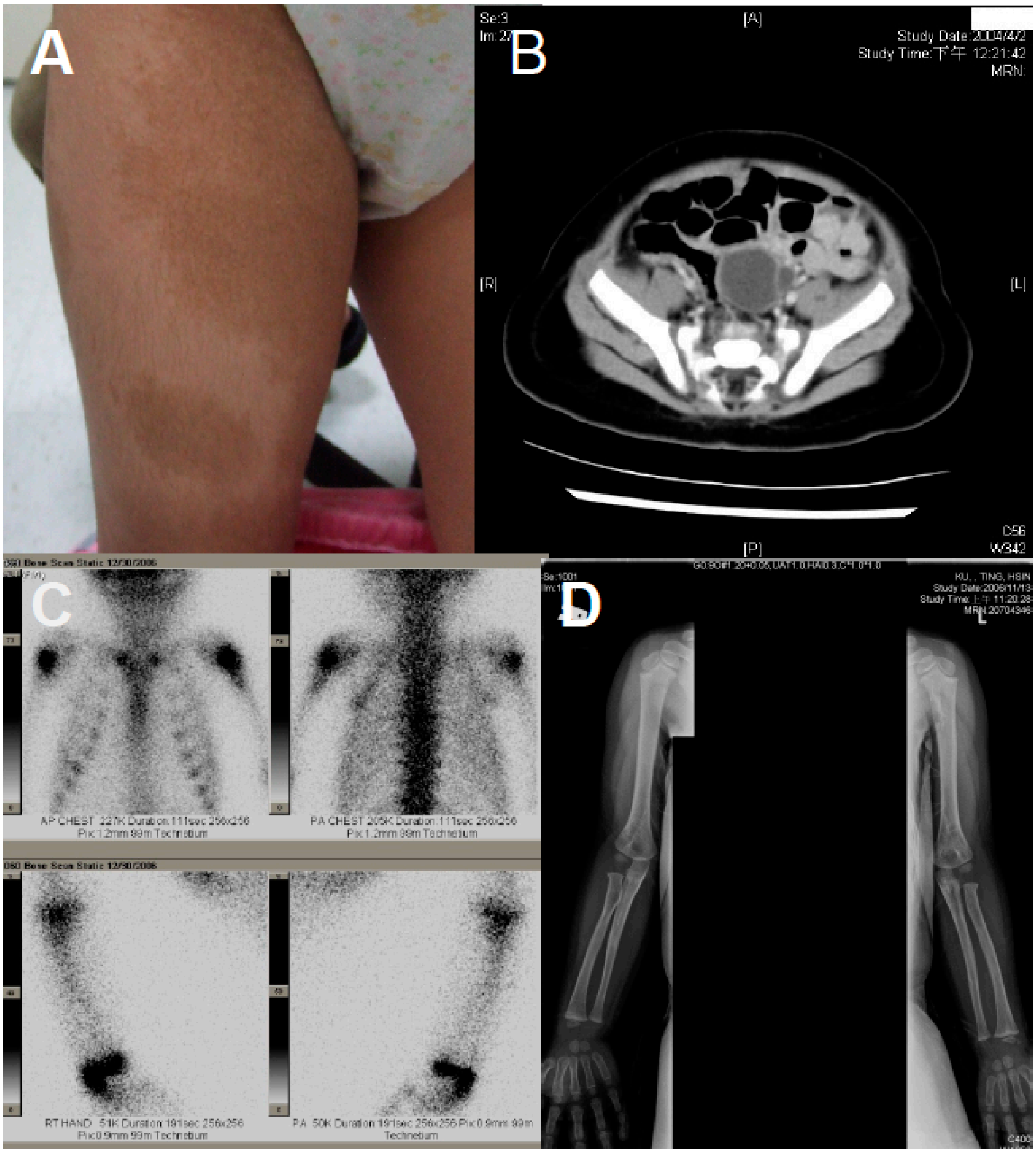

Supplemental Figure S1. Clinical finding in Patient 1. (**A**) Café-au-lait pigmentation visible on her right side thigh. (**B**) Computed tomography of abdomen with contrast enhancement showed one 2.5 × 2.5 cm ovarian cyst at superior aspect of urinary bladder and left adnexa at her age of 3 months. (**C**) Whole body bone scan with Tc-99m methylene diphosphonate showed multiple focal areas of increased uptake of radioactivity involving right occipital area of skull, left proximal humerus, left proximal radius, and left proximal femur at her age of 3-year-1-month old. (**D**) Long bone survey showed radiolucency with marginal sclerosis at left proximal humerus and bilateral proximal humerus at her age of 3-year-7-month old.

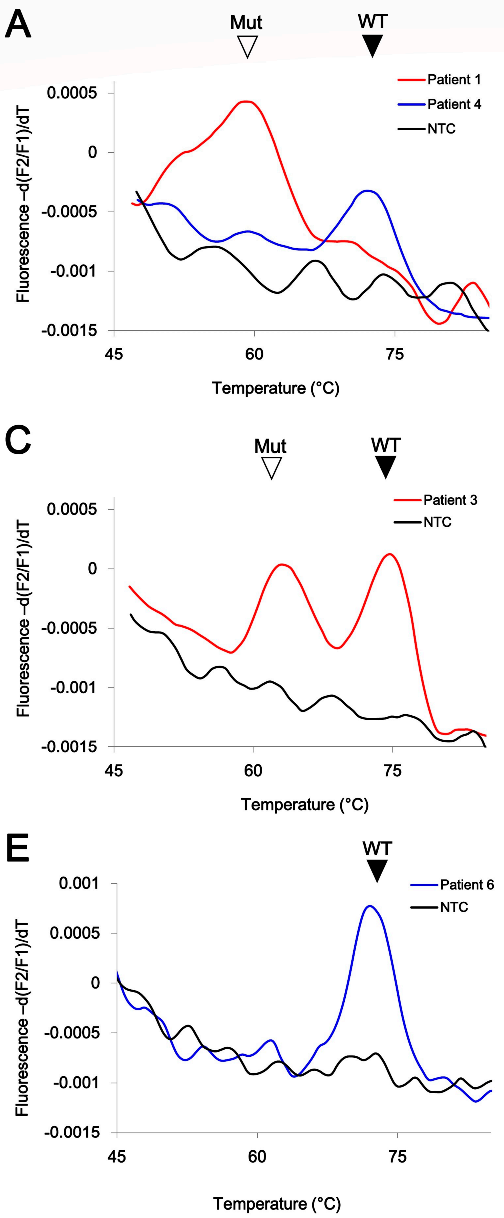

Supplemental Figure S2. The results of PNA probe assay for the six patients. The melting peaks indicate the genotype of the patients. Note that the patients were enrolled at different time, so the results were derived from different experiments. (**A**) Patients 1 and 4, (**B**) Patient 2, (**C**) Patient 3, (**D**) Patient 5, (**E**) Patient 6. The open and the filled arrowheads represent the mutant (Mut) and the wild-type (WT) peaks, respectively. NTC, no template control.
